# An Open-Access Dialysis Membrane-Integrated Microfluidic Device for Generating Drug Exposure Profiles Through Molecular-Weight-Dependent Transport

**DOI:** 10.3390/mi17070835

**Published:** 2026-07-14

**Authors:** Hajime Miyashita, Kenta Shinha, Hiroko Nakamura, Moeno Kadoguchi, Hiroshi Arakawa, Hiroshi Kimura

**Affiliations:** 1 Graduate School of Science and Technology, Tokai University, 4-1-1 Kitakaname, Hiratsuka 259-1292, Kanagawa, Japan; 2 Micro/Nano Technology Center (MNTC), Tokai University, 4-1-1 Kitakaname, Hiratsuka 259-1292, Kanagawa, Japan; 3 Faculty of Pharmaceutical Sciences, Institute of Medical, Pharmaceutical and Health Sciences, Kanazawa University, Kakumamachi, Kanazawa 920-1192, Ishikawa, Japan; 4 Department of Regulatory Science, Graduate School of Pharmaceutical Sciences, Nagoya City University, 3-1 Tanabe-dori, Mizuho-ku, Nagoya 467-8603, Aichi, Japan

**Keywords:** microphysiological system, microfluidic device, dialysis membrane, molecular weight-dependent transport, antibody–drug conjugate

## Abstract

Conventional in vitro assays and many microphysiological systems struggle to generate time-dependent drug exposure profiles because medium replacement simultaneously removes or re-adds drugs in the culture compartment. Here, we developed an Open-access Dialysis Membrane-integrated Microfluidic Device (O-DMiMD) that uses molecular weight-dependent transport across a dialysis membrane to decouple nutrient supply from drug exposure control. The device comprises a cell culture compartment (CCC) and a donor compartment (DC) separated by a dialysis membrane. Transport functions were evaluated using Lucifer Yellow, FITC-dextran, and glucose, followed by drug-response studies using SN-38 and T-DM1 under different medium change conditions. Lucifer Yellow and glucose permeated through the dialysis membrane, whereas FITC-dextran was retained. DC medium change supplied glucose to the CCC and maintained A549/HepG2 co-culture proliferation comparably to direct CCC medium replacement. For SN-38, partial transport to the DC and retention in the CCC generated time-dependent exposure profiles; in A549/HepaRG co-culture, medium change conditions altered A549 viability. For T-DM1, conditions with or without re-addition to the CCC produced different SK-BR-3 responses, suggesting exposure-dependent effects for high-molecular-weight drugs. The O-DMiMD provides an open-access in vitro platform for evaluating drug responses under exposure profiles governed by molecular weights, protein binding, medium changes, and metabolic cell contexts.

## 1. Introduction

The efficacy and toxicity of drugs are greatly influenced not only by the concentration at the time of administration but also by the concentration of the drug to which target tissues and target cells are exposed and the duration of exposure [1]. In vivo, blood drug concentrations continuously change over time due to absorption, distribution, metabolism, and excretion (ADME). Such temporal drug concentration changes are important factors that determine the duration and intensity of drug action on target cells. Exposure metrics such as the area under the concentration–time curve (AUC) and exposure duration have been reported to be associated with therapeutic efficacy and toxicity [2,3]. Therefore, in drug assays, it is important to consider not only the drug concentration at a single time point but also the drug exposure profile in which drug concentrations change over time. Furthermore, drug exposure profiles are strongly influenced not only by temporal drug concentration changes but also by metabolism and protein binding in vivo. In particular, hepatic drug metabolism contributes to drug efficacy and toxicity through drug activation or inactivation [4]. In addition, many drugs exist in the bloodstream in reversible association with plasma proteins. The equilibrium between free and protein-bound drugs influences drug distribution, elimination, and accessibility to target cells [5]. In general, only free drugs are directly involved in cellular activity and membrane permeation. Therefore, protein binding is an important factor that determines effective drug exposure. Since temporal drug concentration changes, metabolism, and protein binding collectively determine drug responses, an evaluation system that can consider these factors is required.

In preclinical studies during the drug discovery process, animal studies (in vivo) and cell-based assays using human-derived cells (in vitro) are conducted to evaluate the pharmacokinetics, efficacy, and toxicity of new drug candidates [6]. Animal studies can evaluate pharmacokinetics and inter-organ interactions at the whole-body level. However, differences in the expression and activity of drug-metabolizing enzymes between experimental animals and humans have been reported [7]. Therefore, there are limitations in directly extrapolating the obtained results to humans. In contrast, in vitro tests using human-derived cells have the advantage of directly evaluating drug responses in human cells. However, conventional static culture systems have difficulty adequately reproducing in vivo mass transport, inter-organ interactions, and temporally changing drug exposure profiles [8]. In addition, it is known that the expression and function of drug-metabolizing enzymes in hepatocyte models decrease depending on the culture conditions. Therefore, it is not easy to evaluate metabolism-mediated drug responses over an extended period [9]. Accordingly, there is a need to build a novel in vitro test system that can reproduce drug exposure profiles and cell–cell interactions similar to those observed in vivo while using human-derived cells.

Against this background, microfluidic devices known as microphysiological systems (MPSs) and organ-on-a-chip platforms have attracted considerable attention [10,11,12,13]. MPSs can build dynamic cell culture environments that are difficult to reproduce in conventional static culture systems because they can control medium perfusion, mass transport, and cell–cell interactions using microfluidic channel structures. In addition, by building co-culture systems composed of multiple cell types, MPSs are expected to be applied to drug response evaluation involving drug metabolism and cell–cell interactions [14]. Maschmeyer et al. built a multiorgan MPS connecting the intestine, liver, skin, and kidney and achieved an ADME evaluation that considered inter-organ interactions [15]. In addition, Vernetti et al. developed a human liver MPS incorporating continuous perfusion and achieved the maintenance of liver function and drug response evaluation under long-term culture conditions [16]. However, in many MPSs, medium change is performed to supply nutrients and remove waste products required for cell maintenance. Although this operation is effective for maintaining the cell culture environment, drugs within the culture compartment are simultaneously removed or re-added. As a result, drug concentrations undergo discrete changes [17]. Consequently, it is difficult to form time-dependent drug exposure profiles in vitro that resemble those observed in vivo. This may lead to overestimation or underestimation of drug efficacy [18]. Therefore, for the application of MPSs to drug assays, a culture platform capable of simultaneously achieving nutrient supply for maintaining the cell culture environment and control of drug exposure profiles is required.

To address this issue, our research group previously developed an MPS incorporating a dialysis membrane, termed the Dialysis Membrane-integrated Microfluidic Device (DMiMD), and reported that it enables molecular weight-dependent transport and drug retention [19]. In this device, low-molecular-weight substances such as glucose are supplied via the dialysis membrane, whereas high-molecular-weight substances are retained within the cell culture chamber. Therefore, the possibility of supplying nutrient molecules required for cell culture while retaining drugs was demonstrated. However, the previously reported device was a closed system. It was difficult to manipulate medium conditions externally during culture. Therefore, it was not sufficient for evaluating drug responses by forming drug exposure profiles in which drug concentrations change over time. Accordingly, an open-access dialysis membrane-integrated microfluidic device is required that can simultaneously achieve drug retention and nutrient supply while allowing easy medium change and sampling during culture.

In this study, we developed an Open-access Dialysis Membrane-integrated Microfluidic Device (O-DMiMD) that can form time-dependent drug exposure profiles while simultaneously achieving drug retention and nutrient supply. Here, “open-access” refers to a physically accessible device structure that enables medium change and sampling. The novelty of this study lies in the integration of a dialysis membrane into an open-access MPS, thereby enabling, within a single platform, both the nutrient supply required to maintain the cell culture environment and the formation of time-dependent drug exposure profiles according to drug properties. This enabled us to construct a platform that facilitates medium change and sampling during culture, which were difficult with the previously reported DMiMD, while allowing a drug-response evaluation that takes drug exposure profiles into account. This device consists of a cell culture compartment (CCC), in which cells are cultured, and a donor compartment (DC), which supplies nutrient molecules, separated by a dialysis membrane. By changing the medium in the DC, low-molecular-weight nutrients such as glucose can be supplied to the CCC via the dialysis membrane. In contrast, proteins, high-molecular-weight drugs, and protein-bound drugs are expected to be readily retained within the CCC. First, the molecular weight-dependent transport characteristics and nutrient supply capability of the device were evaluated using Lucifer Yellow, FITC-dextran, and glucose. Next, drug transport via the dialysis membrane and drug retention within the CCC were evaluated using SN-38, a low-molecular-weight anticancer drug with protein-binding properties. Furthermore, co-culture systems consisting of A549 cells and HepG2 or HepaRG cells were used to investigate the effects of medium change conditions and metabolic function on the response to SN-38. In addition, trastuzumab emtansine (T-DM1), an antibody–drug conjugate (ADC), was used as a model high-molecular-weight drug to evaluate the effects of differences in exposure profiles based on molecular weight-dependent drug retention on cellular responses. Through these investigations, we examined whether this device could form time-dependent drug exposure profiles according to drug properties and medium change conditions and serve as an in vitro drug assay platform capable of evaluating their effects on cellular responses.

## 2. Materials and Methods

### 2.1. Open-Access Dialysis Membrane-Integrated Microfluidic Device (O-DMiMD)

**Figure 1:**
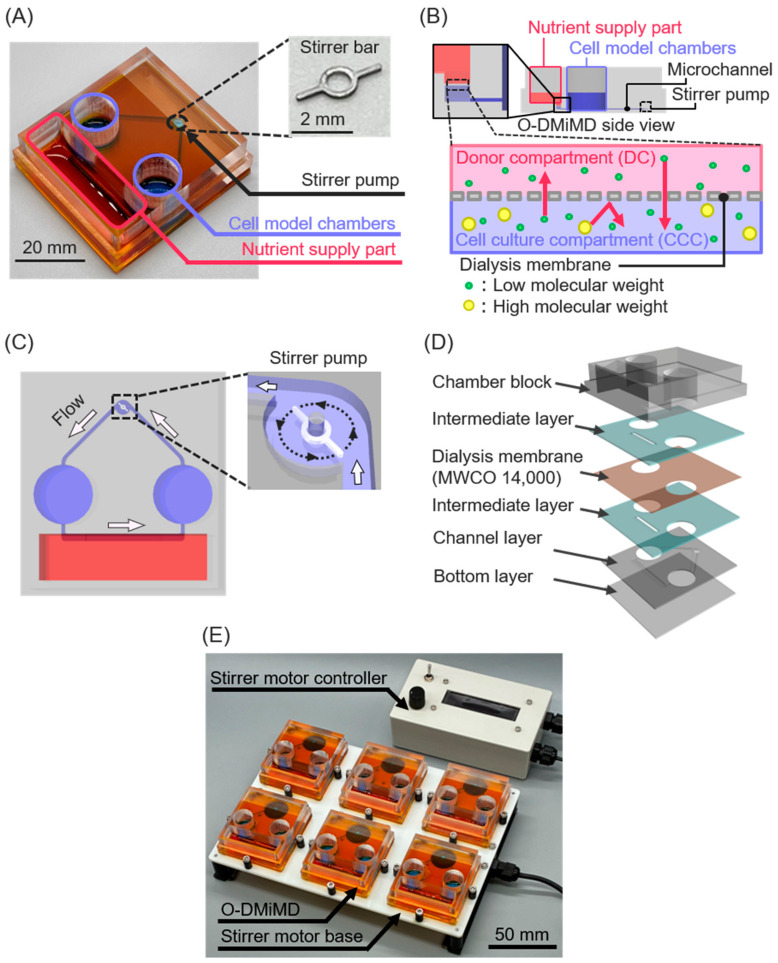
Open-access Dialysis Membrane-integrated Microfluidic Device (O-DMiMD). (**A**) Schematic illustration of the O-DMiMD. The device consisted of an open-access cell culture compartment (cell model chambers, a stirrer pump, and microchannels) and a nutrient supply part (a donor compartment, a dialysis membrane, and a microchannel of the cell culture compartment). (**B**) Side view of the O-DMiMD (**upper**) and a cross-sectional view of the nutrient supply part (**lower**). The (**lower panel**) shows the structure of the nutrient supply part, where the donor compartment and the microchannel in the cell culture compartment are separated by a dialysis membrane. (**C**) Top view of the device. Culture medium in the cell culture compartment was circulated by the stirrer pump. The arrows indicate the direction of medium flow, and the dashed arrow indicates the rotation direction of the stirrer pump. (**D**) Exploded view of the device. The device consisted of a polydimethylsiloxane intermediate layer, a polymethyl methacrylate chamber block, a flow channel layer, and a bottom layer. The dialysis membrane was placed between the intermediate layers, and a stirrer bar was embedded in the flow channel layer. (**E**) Experimental setup. The devices were mounted on a stirrer motor base, enabling simultaneous operation of multiple stirrer pumps.

In this study, we developed an Open-access Dialysis Membrane-integrated Microfluidic Device (O-DMiMD) that simultaneously achieves drug retention and nutrient supply based on molecular weight-dependent transport (Figure 1A). This device was based on the on-chip pump-type MPS developed by Shinha et al. [20] and consisted of a cell culture compartment (CCC) and a donor compartment (DC). The CCC consisted of two cell model chambers, a stirrer pump, and microchannels connecting them. The cell model chamber was an open-access chamber with a diameter of 16 mm, designed to match the geometry of a commercially available 24-well plate. This structure enables easy cell seeding, medium change, and sampling. The volumes of the CCC and DC were 1500 µL and 1750 µL, respectively.

A portion of the microchannel in the CCC and the DC was separated by a dialysis membrane with a molecular weight cut-off (MWCO) of 1.4 × 10^4^ Da and was designated as the nutrient supply part (Figure 1B). The area of the microchannel section in contact with the dialysis membrane was 40 mm^2^. By changing the medium in the DC, low-molecular-weight substances such as glucose passively diffuse into the CCC via the dialysis membrane down their concentration gradient. In contrast, high-molecular-weight drugs and proteins exceeding the MWCO of the dialysis membrane are readily retained within the CCC. This structure was designed to supply low-molecular-weight nutrients from the DC while retaining drugs within the CCC. In addition, the medium in the CCC was circulated by the stirrer pump (Figure 1C).

The O-DMiMD was fabricated using polydimethylsiloxane (PDMS; DOWSILTMSILPOT 184 W/C, Dow Toray, Tokyo, Japan) and polymethyl methacrylate (PMMA; Kanase, Wakayama, Japan). The device consisted of a chamber block (PMMA, 16.0 mm), an intermediate layer (PDMS, 1.0 mm), a dialysis membrane, a microchannel layer (PMMA, 1.0 mm), and a bottom layer (PMMA, 1.0 mm) (Figure 1D). The width and height of the microchannels in the CCC were 1.0 mm and 0.3 mm, respectively. The width of the microchannel section in contact with the dialysis membrane was 2.0 mm. A cellulose dialysis membrane (MWCO 1.4 × 10^4^; F35-7935, Narika, Tokyo, Japan) was used. The PMMA components were fabricated by mechanical machining using a 3D modeling machine (MODELA MDX-50, Roland DG, Shizuoka, Japan). The PDMS intermediate layer was fabricated by cutting a PDMS sheet.

The dialysis membrane was sandwiched between two PDMS intermediate layers and fixed by silane coupling treatment. The PDMS intermediate layers were bonded by oxygen plasma treatment. Polyimide double-sided adhesive tape (760H#25, Teraoka Seisakusho, Tokyo, Japan) was used to bond the other layers. The stirrer pump consisted of a portion of the PMMA microchannel layer and a stainless-steel stirrer bar. The fabricated device was placed on a stirrer motor base. The stirrer bar was rotated at 1500 rpm to circulate the medium counterclockwise within the CCC. The flow rate under this condition was approximately 4.7 µL/min. The stirrer motor base and control unit were manufactured by Microfluidic System Works Inc. (Tokyo, Japan) based on our design. The stirrer motor base was designed to simultaneously drive six devices (Figure 1E). The rotational speed of the stirrer motor was adjusted via current control from the controller.

### 2.2. Cell Culture

Human lung cancer-derived A549 cells (RCB0098, RIKEN Bio Resource Research Center, Ibaraki, Japan) and human breast cancer-derived SK-BR-3 cells (ATCC, Manassas, VA, USA) were used as tumor model cells. In contrast, human liver cancer-derived HepG2 cells (JCRB1054, JCRB Cell Bank, Osaka, Japan) and human hepatocyte-like HepaRG cells (HPR101023, Biopredic International, Saint-Grégoire, France) were used as metabolic model cells. A549, SK-BR-3, HepG2, and HepaRG cells were seeded on Cell desk (MS-92132, Sumitomo Bakelite, Tokyo, Japan) at densities of 1.0 × 10^4^ cells/cm^2^, 1.0 × 10^4^ cells/cm^2^, 2.0 × 10^5^ cells/cm^2^, and 5.5 × 10^4^ cells/cm^2^, respectively. A549 and HepG2 cells were cultured in low-glucose Dulbecco’s Modified Eagle Medium (DMEM; 12320-032, Gibco, Grand Island, NY, USA). The medium was supplemented with 10% fetal bovine serum (FBS; 10270-106, Gibco, Grand Island, NY, USA) and penicillin–streptomycin (161-23181, FUJIFILM Wako Pure Chemical, Osaka, Japan). A549 cells were seeded onto Cell Desk at a density of 1.0 × 10^4^ cells/cm^2^. A549 cells were pre-cultured for 1 day after seeding. HepG2 cells were seeded onto Cell Desk coated with collagen (Cellmatrix^®^ Type I-P, Nitta Gelatin, Osaka, Japan). HepG2 cells were pre-cultured for 3 days after seeding, and the medium was replaced on Day 2. HepaRG cells were cultured using growth medium and differentiation medium prepared by supplementing Basal medium (MIL700, Biopredic International, Saint-Grégoire, France) with Cell Culture Supplement for Growth Medium (ADD710C, Biopredic International, Saint-Grégoire, France) or Cell Culture Supplement for Differentiation Medium (ADD720C, Biopredic International, Saint-Grégoire, France), respectively. HepaRG cells were cultured in growth medium for 14 days and then switched to differentiation medium for an additional 14 days. During the pre-culture period, the medium was changed every 2 days. SK-BR-3 cells were cultured in McCoy’s 5A Medium (16600-082, Gibco, Grand Island, NY, USA). The medium was supplemented with 10% FBS and penicillin–streptomycin. All cells were seeded onto Cell Desk and cultured in an incubator at 37 °C with 5% CO_2_. Pre-culture was performed using commercially available 24-well plates. After completion of the pre-culture period, the cells were introduced into the CCC, along with the Cell Desk, immediately before the start of the experiments.

### 2.3. Molecular Weight-Dependent Permeation Assay

A permeation assay was performed under cell-free conditions to evaluate the molecular weight-dependent transport characteristics via the dialysis membrane in the O-DMiMD. Lucifer Yellow (molecular weight: 521.57 Da) and FITC-dextran (molecular weight: 2.5 × 10^5^ Da) were used in the permeation assay. Each fluorescent compound was introduced into the DC at an initial concentration of 80 µM for Lucifer Yellow and 2 µM for FITC-dextran. Ultrapure water without fluorescent compounds was introduced into the CCC. Subsequently, the CCC was perfused by the stirrer pump.

Samples were collected at 0, 1, 6, 24, 48, 72, 96, 120, and 144 h after the start of perfusion. A 25 µL medium sample was collected from each chamber. Fluorescence intensity was measured using a Grating Microplate Reader (SH-9500, Corona Electric, Ibaraki, Japan). The measured fluorescence intensity was converted to concentration based on calibration curves prepared in advance.

### 2.4. Glucose Measurement

To evaluate the supply capability of low-molecular-weight nutrients through the dialysis membrane, glucose transport from the DC to the CCC was measured. The glucose concentration was measured using a Glucose Assay Kit-WST (G264, Dojindo Laboratories, Kumamoto, Japan). High-glucose DMEM (12430-054, Gibco, Grand Island, NY, USA) was introduced into the DC at a volume of 1750 µL. Low-glucose DMEM was introduced into the CCC at a volume of 1500 µL. The initial glucose concentrations in the DC and the CCC were set at 25.0 mM and 5.6 mM, respectively. Subsequently, the medium in the CCC was perfused by the stirrer pump.

Samples were collected from each chamber at 0, 1, 6, 12, 24, 48, and 72 h after the start of perfusion. A 30 µL volume was collected from each chamber at each sampling point. Samples collected from the DC were diluted 100-fold, whereas samples collected from the CCC were diluted 10-fold. Measurements were performed according to the manufacturer’s protocol. Absorbance was measured at 450 nm using a Grating Microplate Reader. The absorbance values obtained were converted to glucose concentrations using calibration curves prepared in advance. In this assay, the absorbance of WST-formazan generated in proportion to the glucose concentration was used as the measurement index.

### 2.5. SN-38 Measurement

To evaluate the transport behavior of a protein-binding low-molecular-weight drug across the dialysis membrane and its retention within the CCC, the inter-compartmental transport of SN-38 was measured by LC-MS/MS. SN-38 (E0748, Tokyo Chemical Industry, Tokyo, Japan), an anticancer compound, was used as a model drug. Medium without SN-38 (1750 µL) was introduced into the donor compartment. In contrast, medium containing SN-38 (100 nM, 1500 µL) was introduced into the cell culture compartment.

Medium samples were collected on Days 0, 1, 2, 3, 4, 5, and 6. On Days 2 and 4, samples were collected both before and after medium change. A 50 µL volume was collected from each chamber. For sample preparation, 50 µL of acetonitrile containing propranolol (200 nM) as an internal standard was added to each sample. After addition, the samples were vortex-mixed for 5 min. The samples were then centrifuged at 15,000 rpm and 4 °C for 5 min. After centrifugation, the supernatants were collected into MS vials.

LC-MS/MS analysis was performed using an LC-MS8050 triple quadrupole mass spectrometer (Shimadzu, Kyoto, Japan) coupled with an LC-30A system (Shimadzu, Kyoto, Japan). Chromatographic separation was performed using a CAPCELL PAK C18 MG III column (ID 2.0 × 50 mm, Osaka Soda, Osaka, Japan) at 40 °C and a flow rate of 0.4 mL/min under the following step-gradient conditions: 5% solvent B for 1.0 min; linear ramp to 95% solvent B for 3.0 min; then return to the initial conditions in 0.5 min (A, water containing 0.1% formic acid; B, acetonitrile containing 0.1% formic acid). The monitored ion transition was m/z 393.3 > 349.2, and the collision energy (CE) was set to −29 eV. SN-38 concentrations were calculated from calibration curves prepared in advance. The calibration range was 0.1–100 nM, and the lower limit of quantification (LLOQ) was 0.1 nM. Measured concentrations were normalized to the SN-38 concentration in the CCC on Day 0.

The amount of SN-38 was estimated by multiplying the SN-38 concentration in each chamber by the medium volume of the corresponding chamber. Furthermore, the area under the concentration–time curve (AUC) of SN-38 in the CCC was calculated using the trapezoidal rule. The calculated AUC was evaluated as a percentage of the theoretical AUC obtained when the initial SN-38 concentration was maintained constantly for 6 days.

### 2.6. Co-Culture Experiment

In this experiment, A549 cells as tumor model cells and HepG2 cells as metabolic model cells were co-cultured to evaluate whether co-culture conditions within the CCC could be maintained by nutrient supply via the dialysis membrane. The cells were seeded onto Cell Desk at densities of 1.0 × 10^4^ cells/cm^2^ and 2.0 × 10^5^ cells/cm^2^, respectively, and were used after pre-culture. After completion of the pre-culture period, the cells were introduced into the CCC together with the Cell Desk immediately before the start of the experiment. As culture media, high-glucose DMEM (25.0 mM, 1750 µL) was introduced into the DC, and low-glucose DMEM (5.6 mM, 1500 µL) was introduced into the CCC. The culture period was 6 days. Three medium change conditions were compared. The w/o MC (without medium change) condition involved no medium change. The w/MC in CCC (with medium change in the CCC) condition involved medium change in the CCC, similar to conventional in vitro test systems. The w/MC in DC (with medium change in the DC) condition involved changing only the medium in the DC; nutrient supply to the CCC occurred via the dialysis membrane. Medium changes were performed on Days 2 and 4. The cell proliferation rate was evaluated using dehydrogenase activity derived from viable cells as an indicator. The Cell Counting Kit-8 (343-07623, Dojindo Laboratories, Kumamoto, Japan) was used for the measurement. After completion of the co-culture experiment, the Cell Desk seeded with A549 cells was transferred to a commercially available 24-well plate. Absorbance after reagent addition was measured at 450 nm using a Grating Microplate Reader. The measured values were converted to viable cell numbers based on calibration curves prepared in advance. The cell proliferation rate was calculated as the relative viable cell number on Day 6 normalized to that on Day 0. Changes in glucose concentration were also evaluated. Medium samples were collected from each chamber according to the experimental schedule. A 30 µL volume was collected from each chamber at each sampling point. Samples collected from the DC were diluted 100-fold, whereas samples collected from the CCC were diluted 10-fold. Absorbance was measured at 450 nm using a Grating Microplate Reader. The obtained values were converted to glucose concentrations based on calibration curves.

### 2.7. Drug Exposure and Viability Assay

Drug exposure experiments using SN-38 and T-DM1 were conducted to evaluate the effects of different medium change conditions on drug responses. SN-38 was used as a model low-molecular-weight anticancer drug with protein-binding properties. T-DM1 was used as a model antibody–drug conjugate representing high-molecular-weight drugs. SN-38 was used at a concentration of 0.1 µM and dissolved in DMSO. The final concentration of DMSO was 0.1%. T-DM1 was used at a concentration of 0.1 µg/mL and was prepared in PBS.

For the SN-38 exposure study, an A549 monoculture system, an A549/HepG2 co-culture system, and an A549/HepaRG co-culture system were used. For the T-DM1 exposure study, an SK-BR-3 monoculture system was used. Cells were seeded onto Cell Desk and used after pre-culture. Immediately before the start of the experiment, the cells were transferred into the CCC together with the Cell Desk.

For the SN-38 exposure experiment, high-glucose DMEM without SN-38 (1750 µL) was introduced into the DC, whereas low-glucose DMEM containing SN-38 (0.1 µM, 1500 µL) was introduced into the CCC. Two medium change conditions were compared. In the w/MC in CCC condition, the medium in the CCC was changed. In the w/MC in DC condition, the medium in the CCC was not changed, and only the medium in the DC was changed. Medium changes were performed on Days 2 and 4.

For the T-DM1 exposure experiment, medium without T-DM1 (1750 µL) was introduced into the DC, whereas medium containing T-DM1 (0.1 µg/mL, 1500 µL) was introduced into the CCC. Three medium change conditions were compared. In the w/MC in CCC condition, the medium in the CCC was changed. In the w/MC in CCC&DC condition, the media in both the CCC and DC were changed. In the w/MC in DC condition, the medium in the CCC was not changed, and only the medium in the DC was changed. Medium changes were performed on Days 2 and 4. The exposure period was 6 days.

Drug responses were evaluated as cell viability using ATP content as an indicator. The CellTiter-Glo^®^ 2.0 Assay (G9242, Promega Corporation, Madison, WI, USA) was used for the measurements. After completion of drug exposure, the cells were transferred together with the Cell Desk to a commercially available 24-well plate. Luminescence generated after reagent addition was measured using a Grating Microplate Reader. The measured values were converted to ATP content based on calibration curves prepared in advance. Values obtained from the SN-38 exposure study were evaluated as relative cell viability normalized to the untreated control. Values obtained from the T-DM1 exposure study were first calculated as relative cell viability normalized to the untreated control and were subsequently normalized to the value of the w/MC in CCC condition.

### 2.8. Gene Expression Analysis

Because SN-38 is converted to SN-38G, an inactive metabolite, through glucuronidation mediated by UGT1A1, UGT1A1 gene expression levels were evaluated to compare the metabolic characteristics related to the SN-38 inactivation capacity of HepG2 and HepaRG cells. Total RNA was extracted from HepG2 and HepaRG cells using TRIzol (15596-018, Invitrogen, Waltham, MA, USA). The extracted RNA was purified using a Direct-zol RNA Microprep kit (R2062, Zymo Research, Irvine, CA, USA) according to the manufacturer’s instructions. The concentration of purified RNA was evaluated using a NanoDrop Lite spectrophotometer (Thermo Fisher Scientific, Waltham, MA, USA). Purified RNA samples (1 µg each) were reverse-transcribed using an iScript cDNA Synthesis Kit (1708891, Bio-Rad Laboratories, Hercules, CA, USA) according to the manufacturer’s protocol. UGT1A1 gene expression levels were measured using SSo Advanced SYBR Green Supermix (172-5271, Bio-Rad Laboratories, Hercules, CA, USA) and a CFX Connect Real-Time PCR Detection System (Bio-Rad Laboratories, Hercules, CA, USA). PCR amplification was performed after heating at 95 °C for 30 s, followed by 39 cycles consisting of 95 °C for 10 s and 60 °C for 30 s. The primer sequences used for real-time PCR were as follows: GAPDH, forward 5′-TGAAGACGGGCGGAGAGAAA-3′ and reverse 5′-CCAATACGACCAAATCCGTTGAC-3′; UGT1A1, forward 5′-CCTTGCCTCAGAATTCCTTC-3′ and reverse 5′-ATTGATCCCAAAGAGAAAACCAC-3′. GAPDH was used as the housekeeping gene. Relative UGT1A1 expression levels were normalized to GAPDH and calculated using the ΔΔCt method.

### 2.9. Statistical Analysis

Statistical analyses were performed using GraphPad Prism (version 10.3.0, GraphPad Software LLC, Boston, MA, USA). Data are presented as the mean ± standard deviation. Comparisons between two groups were performed using Student’s *t*-test. For comparisons among three groups, homogeneity of variance was assessed using the Brown–Forsythe test. When the assumption of equal variances was satisfied, one-way analysis of variance (one-way ANOVA) followed by Tukey’s multiple comparisons test was performed. When the assumption of equal variances was not satisfied, Brown–Forsythe and Welch ANOVA followed by Dunnett’s T3 multiple comparisons test were performed. Experiments involving two independent factors were analyzed using ordinary two-way ANOVA, followed by Šídák’s multiple comparisons test to compare medium change conditions within each culture condition. No logarithmic, square-root, or other data transformation was applied prior to statistical analysis. A *p*-value of less than 0.05 was considered statistically significant.

## 3. Results and Discussion

### 3.1. Molecular Weight-Dependent Transport and Glucose Supply

To confirm the molecular weight-dependent transport characteristics of the O-DMiMD, the permeation and retention behaviors of two model compounds with different molecular weights were evaluated via the dialysis membrane. In addition, glucose transport between the chambers was evaluated to confirm that low-molecular-weight nutrients could be supplied from the DC to the CCC.

**Figure 2:**
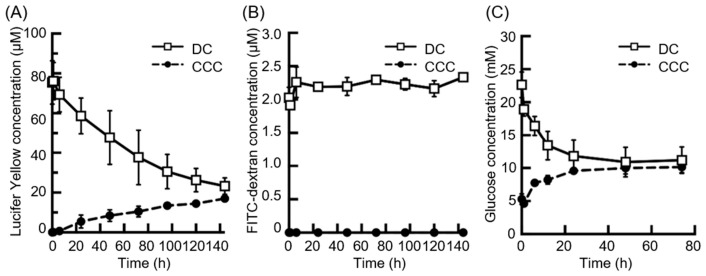
Molecular-weight-dependent transport and glucose supply in the O-DMiMD. (**A**) Time-course changes in Lucifer Yellow concentration (*n* = 4). (**B**) Time-course changes in FITC-dextran concentration (*n* = 4). (**C**) Time-course changes in glucose concentration (*n* = 6). Each compound was introduced into the DC or CCC, and concentration changes in both were measured over time. Data are presented as mean ± standard deviation.

Lucifer Yellow (molecular weight: 521.57 Da) was introduced into the DC as a low-molecular-weight model compound, whereas FITC-dextran (molecular weight: 2.5 × 10^5^ Da) was introduced as a high-molecular-weight model compound. Concentration changes in each chamber were measured over time. The concentration of Lucifer Yellow decreased in the DC and increased in the CCC over time (Figure 2A). In contrast, the concentration of FITC-dextran remained nearly unchanged in the DC and was scarcely detected in the CCC (Figure 2B). These results indicate that Lucifer Yellow was transported from the DC to the CCC via the dialysis membrane, whereas FITC-dextran was retained within the DC. It should be noted that the permeation experiments using Lucifer Yellow and FITC-dextran were conducted in ultrapure water and represent a basic evaluation of molecular weight-dependent transport using model compounds. Therefore, these results do not directly indicate drug transport behavior in the presence of proteins in culture media.

Next, the transport behavior of glucose (molecular weight: 180.16 Da), a low-molecular-weight nutrient essential for cell culture, was evaluated. When high-glucose DMEM was introduced into the DC and low-glucose DMEM into the CCC, the glucose concentration decreased in the DC and increased in the CCC over time (Figure 2C). After 24 h, the difference in glucose concentration between the two chambers became smaller, approaching an equilibrium state. Glucose reached a near-equilibrium state earlier than Lucifer Yellow. Glucose has a molecular weight of 180.16 Da, which is smaller than that of Lucifer Yellow (521.57 Da). Assuming a Stokes–Einstein-type dependence of diffusivity on molecular size, the diffusion coefficient of Lucifer Yellow is estimated to be lower than that of glucose. Although the effective diffusion coefficients through the dialysis membrane were not directly measured in this study and may also be affected by membrane resistance and boundary-layer effects, these differences in molecular size and effective diffusivity likely contributed to the faster approach to equilibrium for glucose than for Lucifer Yellow.

These results demonstrate that the O-DMiMD can supply glucose from the DC to the CCC while retaining high-molecular-weight substances through molecular weight-dependent transport via the dialysis membrane. This finding indicates that the O-DMiMD possesses the fundamental transport characteristics required to supply low-molecular-weight nutrients from the DC while retaining high-molecular-weight substances within the system.

### 3.2. Transport Behavior of SN-38 via the Dialysis Membrane

**Figure 3:**
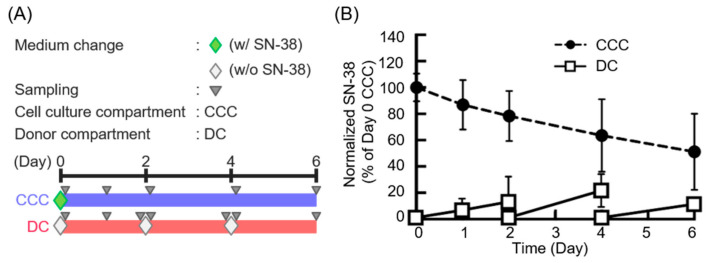
Transport behavior of SN-38 in the O-DMiMD. (**A**) Experimental schedule. SN-38 was introduced into the CCC, and the medium in the DC was changed on Days 2 and 4. (**B**) Time-course changes in SN-38 concentration in the CCC and DC. SN-38 concentrations in the CCC and DC were measured over time. Each value was normalized to the SN-38 concentration in the CCC at Day 0. Data are presented as mean ± standard deviation (*n* = 3).

To clarify the transport behavior of a protein-binding low-molecular-weight drug in the O-DMiMD, SN-38 was used as a model drug to evaluate inter-compartmental transport via the dialysis membrane and retention within the CCC. SN-38 was introduced into the CCC at an initial concentration of 100 nM, whereas medium without SN-38 was introduced into the DC (Figure 3A).

After the introduction of SN-38, its concentration in the CCC gradually decreased, reaching approximately 50% of the initial concentration on Day 6. Concomitantly, SN-38 was detected in the DC. The SN-38 concentration in the DC increased from 0% on Day 0 to approximately 13% before medium change on Day 2 and approximately 21% before medium change on Day 4. After medium changes on Days 2 and 4, the SN-38 concentration in the DC decreased to approximately 0%. However, it increased again due to the transport of SN-38 from the CCC to the DC, and approximately 11% was detected on Day 6 (Figure 3B). In addition, the cumulative amount of SN-38 transported to the DC was generally consistent with the amount lost from the CCC. Furthermore, the cumulative amount of SN-38 removed by medium change in the DC corresponded to approximately 46% of the initial amount loaded into the CCC. These results indicate that a portion of SN-38 was transported from the CCC to the DC via the dialysis membrane.

SN-38 exhibits high binding affinity for plasma proteins, and its protein-binding rate has been reported to be approximately 94–96% [21]. Therefore, a portion of SN-38 in the culture medium is expected to exist in a protein-bound state. Because protein-bound SN-38 has a larger apparent molecular size, it is considered less permeable through the dialysis membrane. In contrast, free SN-38, not bound to proteins, exists as a low-molecular-weight compound and may pass through the dialysis membrane. Therefore, the partial transport of SN-38 to the DC observed in this study may have involved the permeation of free SN-38 through the dialysis membrane.

Despite the transport of a portion of SN-38 to the DC, a substantial fraction of SN-38 remained within the CCC throughout the culture period. The AUC calculated from the SN-38 concentration profile in the CCC corresponded to approximately 72.1% of the theoretical AUC obtained when the initial concentration was maintained for 6 days. SN-38 has been reported to exhibit antitumor activity against A549 cells at a concentration of 53 nM [22]. The SN-38 concentrations observed in the CCC during the present study included this concentration range. These results suggest that, in the O-DMiMD, although a portion of SN-38 was transported to the DC via the dialysis membrane, protein binding may have contributed to the retention of SN-38 within the CCC and partially mitigated excessive drug loss.

It should be noted that free SN-38 and protein-bound SN-38 were not separately quantified in this study. Therefore, the contribution of protein binding to SN-38 retention within the CCC was not directly demonstrated. In addition, changes in SN-38 concentration may have been influenced not only by transport through the dialysis membrane but also by adsorption to device materials and drug degradation. In future studies, the effects of protein binding should be directly examined by comparing permeation behavior under conditions with reduced protein binding, such as serum-free or albumin-free medium, with that under conventional culture medium conditions. Adsorption to device materials and drug degradation should also be investigated to elucidate the mechanism of SN-38 retention in greater detail.

These results suggest that the O-DMiMD can form time-dependent drug exposure profiles characterized by partial permeation and retention rather than complete retention for protein-binding low-molecular-weight drugs. This characteristic may enable the device to evaluate exposure profile-dependent drug responses based on the properties of low-molecular-weight drugs.

### 3.3. Maintenance of Co-Culture Conditions by Nutrient Supply via the Dialysis Membrane

**Figure 4:**
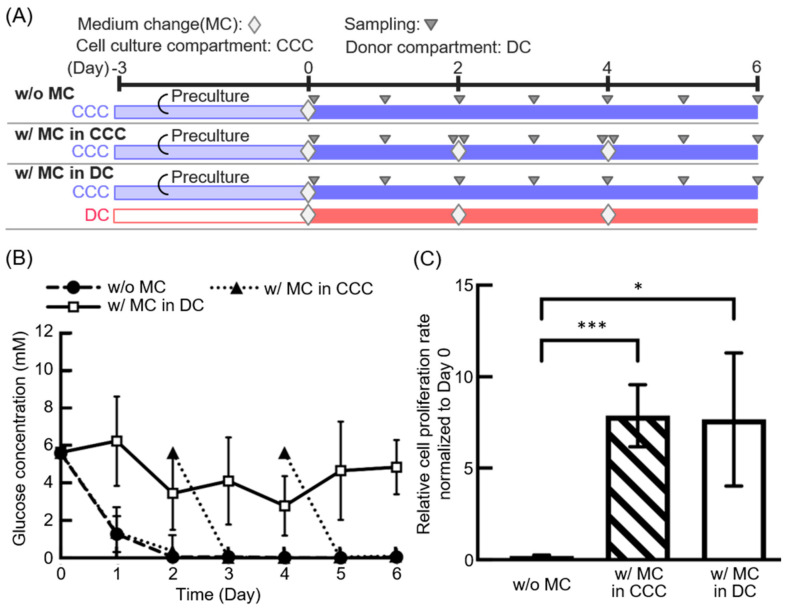
Evaluation of the co-culture environment maintained by nutrient supply through the dialysis membrane. (**A**) Experimental schedule. w/o MC: without medium change; w/MC: with medium change; w/MC in CCC: medium change only in the CCC; w/MC in DC: medium change only in the DC. (**B**) Time-course changes in glucose concentration in the CCC (*n* = 5–6). (**C**) Proliferation rate of A549 cells. Relative cell proliferation rate was calculated as the ratio of Day 6 to Day 0. Statistical analysis was performed using Brown–Forsythe and Welch ANOVA followed by Dunnett’s T3 multiple comparisons test. Data are presented as mean ± standard deviation (*n* = 5–6). * *p* < 0.05 and *** *p* < 0.001.

To evaluate whether co-culture conditions within the CCC could be maintained by medium change in the DC, A549 cells, as tumor model cells, and HepG2 cells, as metabolic model cells, were co-cultured in the CCC. Changes in glucose concentration and cell proliferation rate were evaluated. In this experiment, three medium change conditions were established to compare different nutrient supply strategies (Figure 4A). In the w/o MC condition, neither the DC nor the CCC medium was changed. In the w/MC in CCC condition, only the CCC medium was replaced every 2 days, as in conventional in vitro culture systems. In the w/MC in DC condition, the CCC medium was not changed, and only the DC medium was changed every 2 days. Under all conditions, pre-cultured A549/HepG2 cells were transferred into the CCC together with the Cell Desk, and the medium in the CCC was perfused by the stirrer pump for 6 days.

First, temporal changes in glucose concentration within the CCC were evaluated in the A549-HepG2 co-culture system. Under the w/o MC condition, the glucose concentration decreased over time and was nearly depleted by Day 2. Under the w/MC in CCC condition, glucose concentration decreased over time but was periodically restored to approximately the initial concentration by medium change in the CCC. In contrast, under the w/MC in DC condition, the glucose concentration within the CCC did not decrease markedly throughout the experimental period and remained close to the initial concentration, despite the absence of a direct change in the medium within the CCC (Figure 4B).

Next, the cell proliferation rate of A549 cells was evaluated. Under the w/o MC condition, the cell proliferation rate was the lowest among the three conditions. In contrast, significantly higher cell proliferation rates were observed under the w/MC in CCC and w/MC in DC conditions compared with the w/o MC condition. No significant difference was observed between the w/MC in CCC and w/MC in DC conditions (Figure 4C).

Under the w/o MC condition, glucose within the CCC was almost depleted after Day 2, and the cell proliferation rate was markedly reduced. These results indicate that continuous nutrient supply is required to maintain co-culture conditions of A549 and HepG2 cells. Under the w/MC in CCC condition, glucose was supplied through direct medium change in the CCC, and the cell proliferation rate was maintained. This result demonstrates that the culture environment can be maintained by conventional medium change procedures. In contrast, under the w/MC in DC condition, glucose concentration within the CCC was maintained by medium change in the DC, even though the CCC medium was not directly changed. The cell proliferation rate was also comparable to that observed under the w/MC in CCC condition. These results suggest that glucose was supplied from the DC to the CCC via the dialysis membrane due to the concentration gradient formed between the two compartments.

These results demonstrate that the O-DMiMD can maintain co-culture conditions of A549 and HepG2 cells by supplying glucose through medium change in the DC, even without direct medium change in the CCC. This characteristic is important because it enables partial separation of cell culture maintenance and control of drug exposure profiles. In other words, the device may allow culture maintenance while supplying low-molecular-weight nutrients from the DC without substantially altering drug exposure profiles within the CCC. Therefore, this device may be useful as a co-culture drug assay platform that considers drug exposure profiles.

In this study, glucose concentration was evaluated as a representative indicator of nutrient supply capability via the dialysis membrane. However, the actual cell culture environment is determined not only by glucose but also by multiple factors, including amino acids, lactate, pH, waste products, and oxygen supply. Therefore, further analyses incorporating these metabolic and environmental parameters will be necessary to more comprehensively evaluate the ability of medium change in the DC to maintain the culture environment.

Because this device is compatible with the geometry of a 24-well plate, it is expected to be applicable to parallelization and higher-throughput evaluation using existing cell culture equipment. Although the culture period in this study was limited to 6 days, continuous nutrient supply through the dialysis membrane may enable application to longer-term cultures. Although evaporation was not quantitatively evaluated in this study, no experimental difficulties attributable to evaporation were observed during the 6-day culture period under sufficiently humidified conditions in the incubator.

### 3.4. Responses to SN-38 Under Different Exposure and Metabolic Conditions

**Figure 5:**
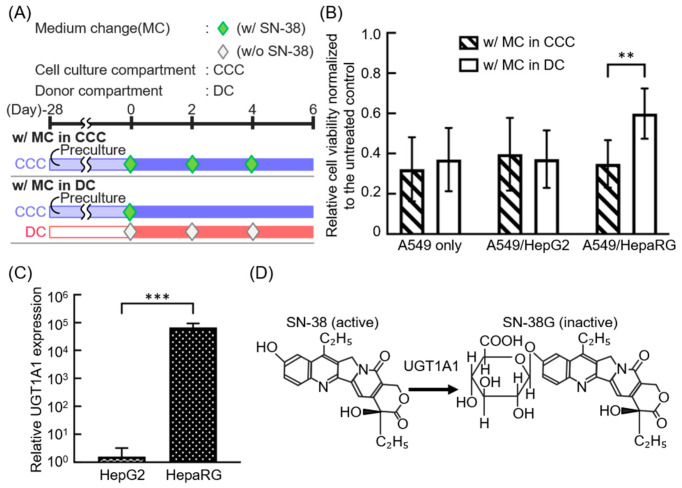
Evaluation of SN-38 efficacy and metabolism-related factors. (**A**) Experimental schedule. SN-38 was exposed for 6 days. w/MC: with medium change; w/MC in CCC: medium change only in the CCC; w/MC in DC: medium change only in the DC. (**B**) Viability of A549 cells evaluated by ATP assay. The evaluation was performed in A549 monoculture and co-culture systems with HepG2 or HepaRG cells. Cell viability was calculated relative to the non-exposed group. Data are presented as mean ± standard deviation (*n* = 6). Statistical analysis was performed using ordinary two-way ANOVA followed by Šídák’s multiple comparisons test. ** *p* < 0.01. (**C**) UGT1A1 gene expression levels in HepG2 and HepaRG cells. Expression levels were normalized to GAPDH. The y-axis is shown on a logarithmic scale. Data are presented as mean ± standard deviation (*n* = 6). Statistical analysis was performed using Student’s *t*-test. *** *p* < 0.001. (**D**) Schematic illustration of the SN-38 metabolic pathway.

To verify the utility of the O-DMiMD as an in vitro drug assay system, drug exposure experiments using the low-molecular-weight anticancer drug SN-38 were conducted. In this experiment, conditions in which only tumor model cells were cultured were compared with conditions in which tumor model cells were co-cultured with metabolic model cells, and the viability of A549 cells after SN-38 exposure was evaluated. A549 cells were used as tumor model cells, whereas HepG2 or HepaRG cells were used as metabolic model cells. Two medium change conditions were established: w/MC in CCC, in which only the CCC medium was changed, and w/MC in DC, in which only the DC medium was changed (Figure 5A). In addition, UGT1A1 gene expression levels were evaluated by real-time PCR to compare the metabolic characteristics related to SN-38 inactivation in HepG2 and HepaRG cells.

Following exposure to SN-38 in the A549 monoculture system, no significant difference in cell viability was observed between the w/MC in CCC and w/MC in DC conditions. Similarly, no significant difference in cell viability was observed between the two medium change conditions in the A549/HepG2 co-culture system. In addition, the viability of A549 cells was comparable between the A549 monoculture system and the A549/HepG2 co-culture system under both medium change conditions. In contrast, in the A549/HepaRG co-culture system, the viability of A549 cells under the w/MC in DC condition was significantly higher than that under the w/MC in CCC condition (Figure 5B). Furthermore, UGT1A1 gene expression levels in HepaRG cells were significantly higher than those in HepG2 cells (Figure 5C).

SN-38 is a topoisomerase I inhibitor that exhibits antitumor activity by inhibiting DNA synthesis as the active metabolite of irinotecan [23]. SN-38 has been reported to undergo glucuronidation by UDP-glucuronosyltransferase 1A1 (UGT1A1) in the liver and be converted to SN-38 glucuronide (SN-38G), an inactive metabolite (Figure 5D) [24]. In addition, SN-38 exhibits high binding affinity to plasma proteins [21]. Because protein-bound SN-38 has a larger apparent molecular size, it is considered less permeable through the dialysis membrane. In contrast, free SN-38 is a low-molecular-weight compound and may be transported to the DC via the dialysis membrane. Therefore, under the w/MC in DC condition, glucose was supplied to the CCC through medium change in the DC, whereas a portion of SN-38 was retained within the CCC. As a result, time-dependent drug exposure profiles may have been formed according to the medium change condition.

In the A549 monoculture system and the A549/HepG2 co-culture system, no significant difference in A549 cell viability was observed between the w/MC in CCC and w/MC in DC conditions. These results indicate that, under these conditions, differences in SN-38 exposure profiles resulting from different medium change methods were not strongly reflected in the cellular response of A549 cells. In addition, the observation that the viability of A549 cells in the A549/HepG2 co-culture system was comparable to that in the A549 monoculture system suggests that the contribution of SN-38 inactivation by HepG2 cells was limited. This interpretation is consistent with the result showing that UGT1A1 expression levels in HepaRG cells were higher than those in HepG2 cells.

In contrast, in the A549/HepaRG co-culture system, the viability of A549 cells under the w/MC in DC condition was significantly higher than that under the w/MC in CCC condition. Under the w/MC in CCC condition, fresh medium containing SN-38 was reintroduced into the CCC during medium change. Therefore, the SN-38 concentration is expected to be periodically restored to a level close to the initial concentration. In contrast, under the w/MC in DC condition, SN-38 within the CCC was not replenished and was affected by transport to the DC via the dialysis membrane and metabolism by HepaRG cells. Consequently, the effective drug exposure may have decreased during the exposure period. Due to these differences in medium change conditions, the total exposure to SN-38 under the w/MC in CCC condition may have been greater than that under the w/MC in DC condition, resulting in a stronger growth inhibitory effect. Furthermore, the higher cell viability observed under the w/MC in DC condition in the A549/HepaRG co-culture system suggests that SN-38 inactivation may have been enhanced in association with the high UGT1A1 expression levels in HepaRG cells.

However, UGT1A1 gene expression is an indirect indicator of the SN-38 inactivation capacity and does not directly reflect actual metabolic activity or SN-38G production. In this study, the concentration of SN-38G in the CCC culture medium after co-culture with HepaRG cells was below the limit of quantification under the analytical conditions used. Therefore, the involvement of SN-38 metabolism proposed in this study is based on the observed UGT1A1 expression and cellular responses and does not directly demonstrate SN-38 metabolism itself. Accordingly, further studies using more sensitive quantification of SN-38G or evaluation of UGT1A1 enzymatic activity will be required to further clarify the relationship between SN-38 metabolism and drug responses.

In any case, these results suggest that different SN-38 exposure profiles were formed in the O-DMiMD depending on the medium change condition and the type of metabolic model cells, and that their effects could be evaluated as cellular responses of A549 cells. In particular, because differences in cell viability dependent on medium change conditions were observed in the A549/HepaRG co-culture system, this device may be useful as an in vitro drug assay platform that considers the interaction between drug exposure profiles and metabolic function.

### 3.5. Response to T-DM1 as a Model Antibody–Drug Conjugate

**Figure 6:**
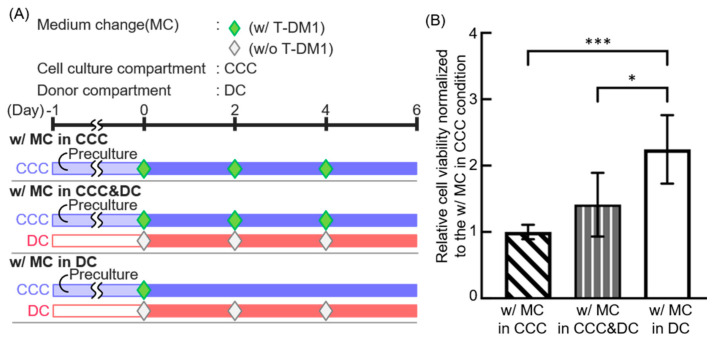
Evaluation of T-DM1 efficacy. (**A**) Experimental schedule. SK-BR-3 cells were monocultured and exposed to T-DM1 for 6 days. w/MC: with medium change; w/MC in CCC: medium change only in the CCC; w/MC in CCC&DC: medium change in both the CCC and DC; w/MC in DC: medium change only in the DC. (**B**) Viability of SK-BR-3 cells evaluated by ATP assay. Cell viability was calculated relative to the non-exposed group and normalized to the value of w/MC in CCC. Data are presented as mean ± standard deviation (*n* = 5–7). Statistical analysis was performed using one-way ANOVA followed by Tukey’s multiple comparisons test. * *p* < 0.05 and *** *p* < 0.001.

To evaluate the effect of drug retention based on molecular weight-dependent transport on the drug response of antibody–drug conjugates (ADCs), which are high-molecular-weight drugs, T-DM1 exposure experiments were conducted using the O-DMiMD. In this experiment, SK-BR-3 cells, a HER2-overexpressing human breast cancer cell line, were used as tumor model cells, and cell viability after exposure to trastuzumab emtansine (T-DM1), a representative ADC targeting HER2, was evaluated. Three medium change conditions were established (Figure 6A). In the w/MC in CCC condition, only the CCC medium was changed. In the w/MC in CCC&DC condition, the media in both the CCC and DC were changed. In the w/MC in DC condition, the CCC medium was not changed, and only the DC medium was changed.

Following exposure to T-DM1, no significant difference in SK-BR-3 cell viability was observed between the w/MC in CCC and w/MC in CCC&DC conditions. In contrast, the viability of SK-BR-3 cells under the w/MC in DC condition was significantly higher than that under the w/MC in CCC and w/MC in CCC&DC conditions (Figure 6B).

ADCs are high-molecular-weight therapeutic agents that selectively deliver cytotoxic payloads to target tumor cells through monoclonal antibodies [25,26]. Their efficacy depends on antibody target recognition, cellular internalization, linker or antibody degradation, and subsequent payload release [27,28]. In addition, ADCs are high-molecular-weight drugs that exhibit longer circulation half-lives than low-molecular-weight drugs [29]. After binding to HER2, T-DM1 is internalized by endocytosis into tumor cells and subsequently degraded in lysosomes, releasing DM1 [30]. Because T-DM1 has a molecular weight far exceeding the MWCO of the dialysis membrane, its transport to the DC via the dialysis membrane is expected to be limited, resulting in preferential retention within the CCC.

The absence of a significant difference in cell viability between the w/MC in CCC and w/MC in CCC&DC conditions suggests that the transport of T-DM1 to the DC was limited and that SK-BR-3 cells were exposed to similar levels of T-DM1 under both conditions. In both conditions, fresh medium containing T-DM1 was reintroduced into the CCC during medium change. Therefore, the effective exposure level within the CCC was likely maintained at a relatively high level. In contrast, under the w/MC in DC condition, T-DM1 within the CCC was not replenished. Consequently, the effective exposure level may have decreased compared with the w/MC in CCC and w/MC in CCC&DC conditions. The significantly higher viability of SK-BR-3 cells observed under this condition suggests that the effect of T-DM1 on SK-BR-3 cells may have been reduced during the exposure period due to decreases in the effective concentration caused by cellular uptake and degradation of T-DM1.

However, in this study, the concentration of T-DM1 in the CCC and DC, intracellular uptake of T-DM1, and the concentration of released DM1 were not directly quantified. In addition, because the drug concentration in the CCC was not measured over time during the drug efficacy experiments, the direct relationship between the drug exposure profile and cellular responses was not demonstrated. Therefore, the retention behavior of T-DM1, changes in the effective exposure level, and their direct relationship with cellular responses were not demonstrated in this study. In future studies, the mass balance, drug exposure profiles, and drug responses of T-DM1 should be evaluated more comprehensively by quantifying the concentration of T-DM1 in the CCC and DC, intracellular uptake of T-DM1, and the concentration of released DM1, together with time-course measurement of the drug concentration in the CCC during the drug efficacy experiments.

These results suggest that the O-DMiMD can form different drug exposure profiles for the high-molecular-weight drug T-DM1 depending on the medium change condition. In particular, because different cellular responses were observed between conditions with and without re-addition of T-DM1 to the CCC, this device may be applicable to in vitro drug assays of high-molecular-weight drugs such as ADCs while considering both drug retention and medium change conditions.

## 4. Conclusions

In this study, we developed an O-DMiMD that can generate time-dependent drug exposure profiles while simultaneously achieving drug retention and nutrient supply through molecular-weight-dependent transport across a dialysis membrane. This device consists of the CCC and the DC separated by a dialysis membrane and is characterized by its ability to supply low-molecular-weight nutrients from the DC without direct medium change in the CCC. Evaluation using model compounds demonstrated that Lucifer Yellow and glucose were transported through the dialysis membrane, whereas FITC-dextran was retained. In addition, it was confirmed that glucose could be supplied to the CCC through medium change in the DC, thereby maintaining co-culture conditions of A549 and HepG2 cells. Furthermore, in experiments using SN-38, a protein-binding low-molecular-weight anticancer drug, a portion of SN-38 was transported to the DC via the dialysis membrane, whereas SN-38 concentrations within a pharmacologically active range were maintained in the CCC for a certain period. In the A549/HepaRG co-culture system, significant differences in cell viability were observed depending on the medium change condition, suggesting that drug exposure profiles and the characteristics of metabolic model cells may influence cellular responses. In addition, experiments using the ADC T-DM1 demonstrated different cellular responses between conditions with and without re-addition of T-DM1 to the CCC, suggesting that medium change conditions may influence effective exposure levels, even for high-molecular-weight drugs.

The novelty of this study lies in the integration of a dialysis membrane into an open-access MPS, thereby simultaneously achieving the nutrient supply required to maintain the cell culture environment and to generate drug exposure profiles according to drug properties on a single platform. In particular, the ability to alter drug exposure profiles within the CCC based on medium change conditions and molecular weight-dependent transport while supplying low-molecular-weight nutrients from the DC represents a unique feature that differs from conventional in vitro culture systems employing complete medium replacement. However, because SN-38G and T-DM1 concentrations within the CCC, intracellular uptake levels, and released DM1 concentrations were not directly quantified in this study, additional investigations are required to further clarify the relationship between drug disposition and cellular responses. Taken together, these findings suggest that the O-DMiMD is a useful novel in vitro drug assay platform capable of forming drug exposure profiles according to molecular weight, protein-binding properties, medium change conditions, as well as the characteristics of metabolic model cells, and of evaluating their effects as cellular responses.

## Data Availability

The original contributions presented in this study are included in the article. Further inquiries can be directed to the corresponding author.
